# Supplementary material for: Augmenting interpretable models with large language models during training
Source: Nat Commun. 2023 Nov 30;14:7913. doi: 10.1038/s41467-023-43713-1 (PMC10689442; doi:10.1038/s41467-023-43713-1)
Supplement: Supplementary file 1 — Supplementary Information [file 41467_2023_43713_MOESM1_ESM.pdf]

Supplementary Information for "Augmenting Interpretable Models with Large Language Models during Training"

1 Supplement Aug-Linear

Varying Aug-Linear settings for main datasets By default (Table 2), we use the final embedding layer of the model (and average it over the sequence length to get a fixed size vector), but Table 2 also shows results using the *pooler output* layer of the BERT model. The choice of layer (i.e. final embedding layer versus pooler output) does not seem to make a large difference in the final performance results. Table 2 also shows one variation of the model (*BERT finetuned (noun chunks)*) where rather than training on all ngrams, the model is fit to only noun-phrases extracted by spaCy's dependency parser [39]. This results in a performance drop across the datasets, suggesting that these noun-phrases alone are insufficient to perform the classification task.

We also run an experiment where we extract embeddings using Instructor ([93], `hkunlp/instructor-xl`), which allows giving a contextual prompt for each dataset. We also use test 2 sizes of GPT-2 [94] and the 7-billion parameter LlaMA model [95].

Evaluating zero-shot accuracy with language models To measure generalization ability, we evaluate explanations based on accuracy as a prompt for other models. Accuracy is computed following [1, 96]: using exact matching with beam search, a beam width of 4, and a length penalty of $\alpha = 0.6$. For sentiment evaluation, we use each prompt with the template *Input: "${input}"{prompt}*.[1] We use *positive* and *negative* as positive and negative labels and require the LLM to rank the two options. Human-written prompts are adapted to this template from open-source prompts available through PromptSource [27].

1.1 Test-time tradeoffs between accuracy and interpretability/speed

The ability to effectively generalize to unseen tokens in Fig 4C/D raises the question of whether one can vary the order of ngrams used *at test-time*, to get a tradeoff between accuracy and interpretability (i.e. how many features are used to make a prediction). Depending on the relative importance of accuracy and interpretability for a given problem, one may select to use a different number of features for testing. Fig 2 suggests that this is feasible.

Fig 2A shows the prediction performance when compressing the Aug-Linear model (fit using 4-grams and finetuned BERT) by setting the coefficients with the smallest magnitudee to zero. Some models require only a few coefficients to perform well and some models (e.g. the *Emotion* and *Financial phrasebank* models) predict more accurately when using less than 50% of the original coefficients. Fig 2B it shows the accuracy of the same models in Fig 2A, as the order of ngrams used *only for testing* is varied. As the number of features used for testing increases, the performance tends to increase but interpretations become more difficult.

Fig 3 characterizes the full tradeoff between the number of ngrams used for fitting versus testing for all datasets. Generally, the best performance is achieved when the same number of ngrams is used for training and testing (the diagonal). Performance tends to degrade significantly when fewer ngrams are used for testing than training (lower-left).

[1] In initial experiments, we find that performance drops significantly when learning a prompt that comes *before* the input.

Supplementary Table 1: Table of pre-trained models with unique huggingface identifiers. All models are used through huggingface [88], and linear/tree baselines are fit with scikit-learn [40] and imodels [54].

	BERT
Base (no finetuning)	`bert-base-uncased` [3]
Emotion	`nateraw/bert-base-uncased-emotion`
Financial phrasebank	`ahmedrachid/FinancialBERT-Sentiment-Analysis` [89]
Rotten tomatoes	`textattack/bert-base-uncased-rotten_tomatoes` [90]
SST2	`textattack/bert-base-uncased-SST-2` [90]
	**DistilBERT**
Base (no finetuning)	`distilbert-base-uncased` [87]
Emotion	`aatmasidha/distilbert-base-uncased-finetuned-emotion`
Financial phrasebank	`yseop/distilbert-base-financial-relation-extraction` [91]
Rotten tomatoes	`textattack/distilbert-base-uncased-rotten-tomatoes` [90]
SST2	`distilbert-base-uncased-finetuned-sst-2-english`
	**RoBERTa** [92]
Emotion	`bhadresh-savani/roberta-base-emotion`
Financial phrasebank	`abhilash1910/financial_roberta`
Rotten tomatoes	`textattack/roberta-base-rotten-tomatoes` [90]
SST2	`textattack/roberta-base-SST-2` [90]

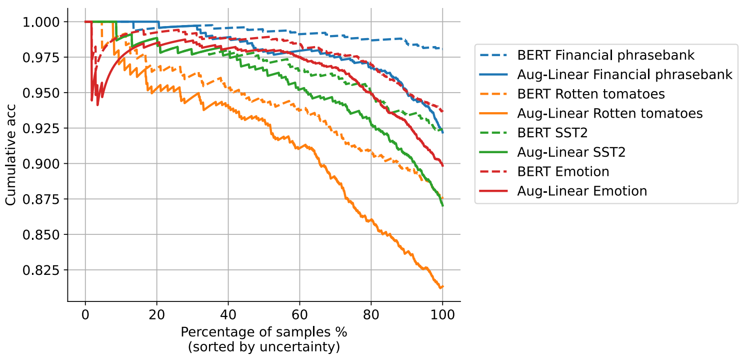

Supplementary Figure 1: Model performance decreases with increasing model uncertainty. Cumulative validation accuracy decreases as more uncertain samples (based on the model's predicted probability) are added.

1.2 Comparison with post-hoc feature importance

The coefficients learned by Aug-Linear often differ from importances assigned by post-hoc feature-importance methods. Aug-Linear learns a single coefficient for each ngram across the dataset, allowing for auditing/editing the model with visualizations such as Fig 4. In contrast, popular methods for post-hoc feature importance, such as LIME [28] and SHAP [29] yield importance scores that vary based on the context in each input. This can be useful for debugging complex nonlinear models, but these scores (i) are approximations, (ii) must summarize nonlinear feature interactions, and (iii) vary across predictions, making transparent models preferable whenever possible.

Fig 4 shows an example of the Aug-Linear coefficients for the SST2 model from Fig 4 for different ngrams when making a prediction for the phrase *not very good*. While Aug-Linear yields scores for each subphrase that match human judgement (as seen in Fig 4B/D), posthoc feature importance methods summarize the interactions between different ngrams into individual words, potentially making interpretation difficult. Scores are rescaled to be between -1 and 1 to make them comparable. See Aug-Linear scores for many top-interacting phrases in Fig 5.

Supplementary Table 2: Generalization accuracy varies depending on the model used to extract embeddings. Finetuning the embedding model improves Aug-Linear performance, using a BERT model seems to outperform a DistilBERT model, and the layer used to extract embeddings does not have too large an effect. Larger models do not necessarily improve performance. †Single-layer finetuned is not an Aug-Linear model, but a black-box fintuned model. Top two methods are bolded in each column. Averaged over 3 random dataset splits; all standard errors are below 0.37.

	Model	FPB	RT	SST2	Emotion
Aug-Linear	BERT finetuned	**92.8**	81.6	86.9	**89.5**
	BERT finetuned (pooler output)	**93.5**	81.3	**87.8**	**89.8**
	BERT finetuned (noun chunks)	87.9	79.7	84.1	87.1
	BERT	84.1	78.1	82.8	67.1
	BERT (pooler output)	82.7	78.5	80.7	58.0
	DistilBERT finetuned	85.8	78.5	81.7	68.8
	DistilBERT	81.7	79.8	86.8	87.5
	RoBERTa finetuned	77.8	**83.6**	**89.1**	88.5
	LLaMA (7B)	82.1	**82.0**	81.7	73.4
	Instructor	76.5	80.0	84.7	71.0
	GPT-2 (1.5B)	76.0	81.5	78.8	68.0
	GPT-2 (124M)	71.9	76.2	71.4	49.6
Interpretable baseline	TF-IDF	84.9	75.9	83.4	89.2
Black-box baseline	†BERT single-layer finetuned	86.3	81.3	85.3	64.8

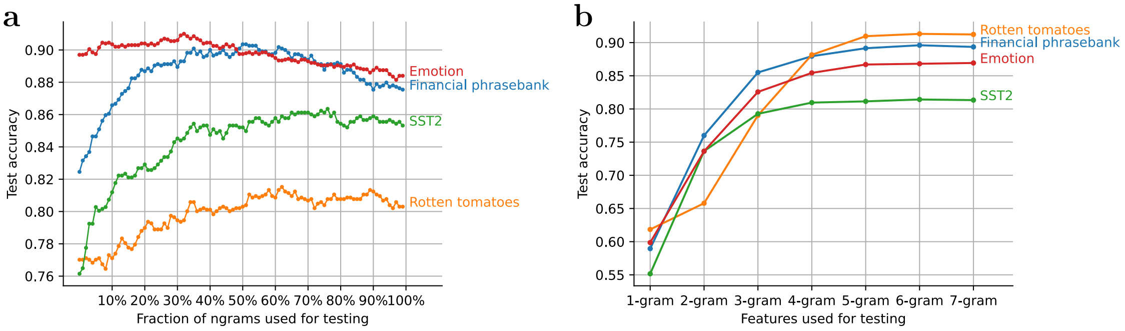

Supplementary Figure 2: Aug-Linear performance when varying the ngrams used for *testing*. **(a)** Performance when removing the smallest coefficients from an Aug-Linear model. **(b)** Performance when varying the order of ngrams used for testing.

Summing embeddings meaningfully captures interactions One potential concern with the Aug-Linear model is that it may fail to learn interactions since it simply sums the embeddings of individual ngrams, and the language model extractor may not sufficiently capture interactions in its embedding space. To investigate this concern, we first identify bigrams that involve interaction by fitting a unigram bag-of-words model and a bigram bag-of-ngrams model to *SST2*. We then use these two models to select the 10 bigrams for which the bigram coefficient is farthest from the sum of the coefficients for each unigram.

Fig 5 shows the resulting bigrams containing interactions. For each bigram, it shows the Aug-Linear learned coefficient (i.e. the contribution to the prediction $w^T \phi(x_i)$) for the bigram (gray bar) along with each

Supplementary Table 3: Generalization accuracy for multiclass datasets and across tokenizers. AG-News has 4 classes, DBPedia-14 has 14 classes, and TREC has 6. Top two methods are bolded in each column. Averaged over 3 random dataset splits; all standard errors are below 0.25.

	Model	AG-News [97]	DBPedia-14 [98]	TREC [99]
Aug-Linear (NLTK [100] tokenization)	BERT finetuned	**90.6**	97.9	**94.2**
	BERT	88.8	96.9	90.0
	GPT-2 (124M)	89.6	94.1	80.2
Aug-Linear (Spacy [39] tokenization)	BERT finetuned	**90.6**	**98.0**	**94.4**
	BERT	88.8	96.9	89.0
	GPT-2 (124M)	86.1	91.1	79.6
	GPT-2 (1.5B)	86.4	96.6	90.2
	Instructor	89.2	96.4	88.0
	LlaMA (7B)	86.8	97.0	93.2
Interpretable baseline	TF-IDF	87.9	95.7	87.4
Black-box baseline	BERT single-layer finetuned	**90.4**	**98.4**	92.8

of its constituent unigrams (blue and orange bars). It is clear that the bigram coefficient is not the simple naive sum of the unigram coefficients (dashed black bar), and the learned coefficients make intuitive sense, suggesting that this Aug-Linear model has successfully learned interactions.

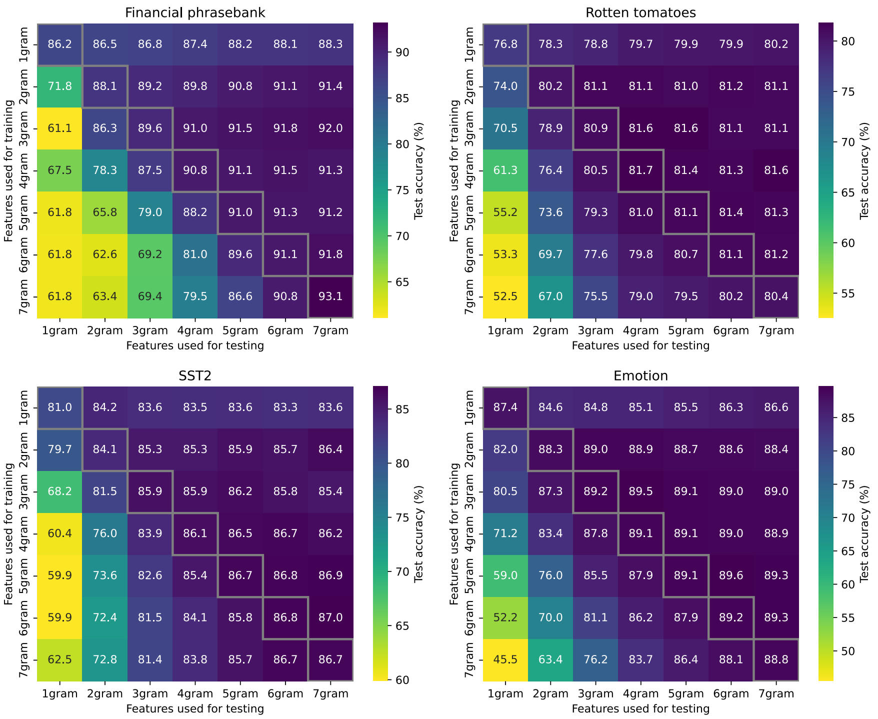

Supplementary Figure 3: Varying the order of ngrams used for training and testing across each of the five datasets in Table 1. Some models (i.e. rows) perform reasonably well as the order of ngrams used for testing is varied, potentially enabling a test-time tradeoff between accuracy and interpretability. Generally, using higher-order ngrams during testing improves performance and testing with less ngrams than used for training hurts performance considerably.

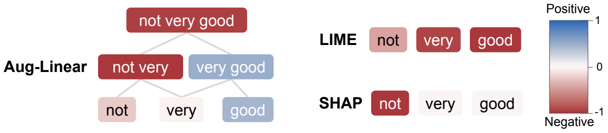

Supplementary Figure 4: Comparison to post-hoc methods. Comparing Aug-Linear ngram coefficients (left) to word-level feature importances from posthoc methods (right): LIME and SHAP.

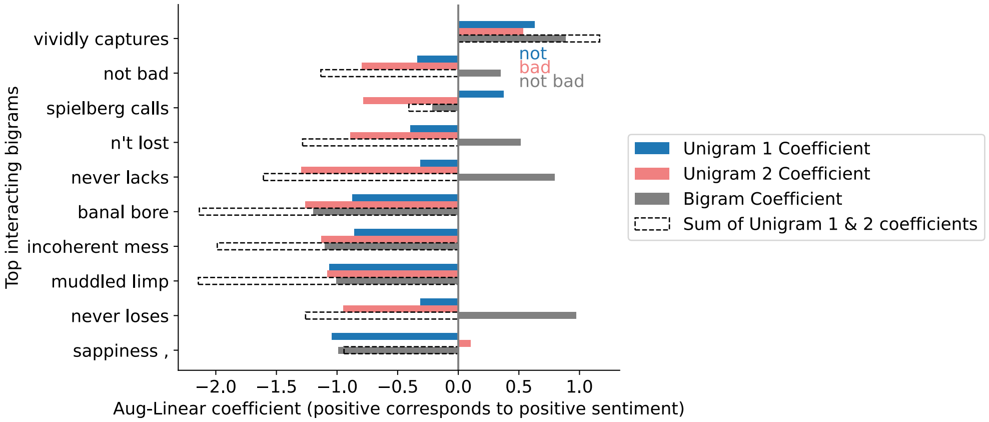

Supplementary Figure 5: Depiction of interactions. Aug-Linear accurately learns interactions rather than simply summing the contributions of individual unigrams.

Supplementary Table 4: Model size comparisons for models studied here. LLMs are generally much larger than Aug-Linear models. Aug-Linear size depends on the number of unique ngrams in the data it is fit to (see Table 1); here we show the model size when the Aug-Linear is fit to just the train set, or to bothe the train and test set jointly. We show sizes for the datasets which yield the smallest and largest Aug-Linear models. Both LLMs and Aug-Linear models can be compressed using techniques such as quantization. Aug-Linear inference for the same parameter count as an LLM is considerably faster, as it requires only dictionary lookups of ngrams and a single summation rather than cascading matrix multiplications and nonlinearities.

	Model	Number of parameters
	LlaMA [95]	7B
	BERT (`bert-base-uncased`)	110M
	DistilBERT (`distilbert-base-uncased`)	66M
Train set	Aug-Linear (Emotion, trigram)	201K
	Aug-Linear (FPB, trigram)	40K
	Aug-Linear (Emotion, bigram)	106K
	Aug-Linear (FPB, bigram)	28K
	Aug-Linear (Emotion, unigram)	15K
	Aug-Linear (FPB, unigram)	7.1K
Train + test set	Aug-Linear (Emotion, trigram)	395K
	Aug-Linear (FPB, trigram)	75K
	Aug-Linear (Emotion, bigram)	202K
	Aug-Linear (FPB, bigram)	51K
	Aug-Linear (Emotion, unigram)	26.5K
	Aug-Linear (FPB, unigram)	11.4K

2 Supplement Aug-Tree

Table 6 explores different variations of Aug-Tree. The top row shows learning a single tree with Aug-Tree using its default parameters, achieving the best performance across the datasets. Table 6 shows results for different algorithmic choices, such as replacing the generic prompt with a dataset-specific one (*Aug-Tree (Contextual prompt)*), and searching for new keyphrases using 5 CART features instead of one (*Aug-Tree (5 CART features)*). We also consider preprocessing the data differently, using *Stemming* (with the Porter Stemmer) or using *Trigrams*, rather than bigrams.

One major variation we study is using LLM embeddings to find keyphrases, rather than querying via a prompt (*Aug-Tree (Embeddings*). Specifically, we consider expanding keywords by finding the keyphrases that are closest in embedding space (measured by euclidean distance) to the original keyphrase. This option may be desirable computationally, as it may require a smaller LLM to compute effective embeddings (e.g. BERT [3]) compared to a larger LLM required to directly generate relevant keyphrases (e.g. GPT3 [1]). However, finding closest embeddings requires making more calls to the LLM, as embeddings must be calculated and compared across all ngrams in X_{text}.

Supplementary Table 5: Metadata on keyphrase expansions. Results are averaged over keyphrases found in the 4 text-classification datasets in Table 1 when fitting a 40-tree bagging ensemble. The LLM is queried for 100 expansion candidates, but due to imperfect LLM generations, only 91.6 candidates are generated on average. After deduplication (converting to lowercase, removing whitespaces, etc.), only 83.3 candidates remain. Screening removes almost all candidates, leaving only 0.8 candidates on average.

# Expansion candidates (Before deduplication)	# Expansion candidates	# Expansions (After screening)
91.6±0.7	83.3±0.8	0.8±0.1

Supplementary Table 6: Performance (ROC AUC) for variations of Aug-Tree. Values are averaged over 3 random dataset splits; error bars are standard error of the mean (many are within the points).

	Emotion	Financial phrasebank	Rotten tomatoes	SST2
**Aug-Tree**	**0.680 ±0.029**	**0.825 ±0.006**	**0.622 ±0.007**	**0.673 ±0.008**
Aug-Tree (BERT Embeddings)	0.599 ±0.008	0.776 ±0.018	0.600 ±0.011	0.663 ±0.002
Aug-Tree (Contextual prompt)	0.667 ±0.011	0.820 ±0.004	0.627 ±0.008	0.669 ±0.005
Aug-Tree (5 CART features)	0.711 ±0.039	0.730 ±0.026	0.608 ±0.009	0.674 ±0.003
Aug-Tree (Stemming)	0.640 ±0.019	0.520 ±0.016	0.625 ±0.004	0.679 ±0.005
Aug-Tree (Trigrams)	0.676 ±0.030	0.826 ±0.006	0.619 ±0.010	0.669 ±0.006
CART	0.574 ±0.002	0.775 ±0.005	0.599 ±0.005	0.636 ±0.002
ID3	0.573 ±0.004	0.795 ±0.010	0.589 ±0.002	0.638 ±0.009

Supplementary Table 7: Performance (Accuracy) for Aug-Tree and Aug-Tree Ensemble. Values are averaged over 3 random dataset splits; error bars are standard error of the mean (many are within the points). **Emotion* and *Financial phrasebank* results are not directly comparable to Table 2, as they have been modified for binary classification.

	Emotion*	Financial phrasebank*	Rotten tomatoes	SST2
Aug-Tree	0.637 ±0.045	0.818 ±0.014	0.613 ±0.009	0.571 ±0.018
Aug-Tree Ensemble	0.800 ±0.008	0.848 ±0.006	0.619 ±0.004	0.614 ±0.016

3 Supplement fMRI experiment details

This section gives more details on the fMRI experiment analyzed in Sec 2.5; for more scientific details see the original study [22]. Sec 2.5 analyzes data from one human subject (`UTS03`) in the original study, as the subject listened to approximately hours of narrative speech from the Moth Radio Hour, which consists of short autobiographical stories. The subject underwent fMRI scanning as they listened, yielding an fMRI volume brain scan consisting of 95,556 voxels roughly every two seconds.

The individual voxel models described in Sec 2.5 are each fit to 9,461 training points, each corresponding to a different time point (after accounting for various preprocessing steps, such as trimming the beginning and end of the sequence). They are evaluated on 291 volumes which come from a narrative story that was not seen during training.

Fig 6 shows the generalization performance of the model for each voxel, measured by the correlation between the predicted response and the measured response. Fig 5 shows the performance difference between the Aug-Linear model and the BERT baseline.

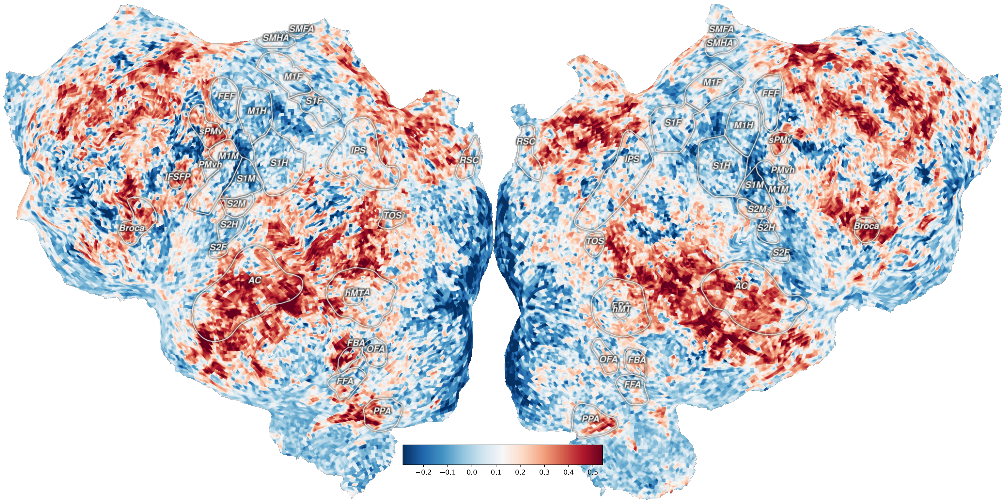

Supplementary Figure 6: Generalization performance for individual-voxel models. Performance is measured by correlation between the predicted response and the measured response on the held-out test set. Some regions are very poorly predicted (blue), but many voxels can be predicted quite well (red).

Supplementary Table 8: fMRI prediction performance using different methods. Eng1000 is a linear word-embedding baseline similar to word2vec which has been used in the neuroscience literature [30].

Model	Order of ngram	ρ	ρ (top 1%)	ρ (top 5%)
Eng1000	1	0.041	0.529	0.439
GloVe	1	0.044	0.521	0.426
BERT	5	0.022	0.386	0.302
BERT	10	0.035	0.457	0.365
BERT	20	0.053	0.524	0.429
Aug-Linear (BERT)	5	0.061	0.583	0.489
**Aug-Linear (BERT)**	**10**	**0.062**	**0.586**	**0.489**
Aug-Linear (BERT)	20	0.056	0.566	0.470
